# Unveiling the Mechanistic Impact of Mutations F2004C/V in the ROS1 Kinase Domain

**DOI:** 10.1021/acsomega.5c00072

**Published:** 2025-05-30

**Authors:** Juliana F. Vilachã, Farhan Ul-Haq, Geert Vandeweyer, Siewert-Jan Marrink

**Affiliations:** † School of Life Sciences & Department of Chemistry, 2707 University of Warwick—Coventry Campus, Gibbet Hill Campus, CV4 7AL, Coventry CV4 7AL, U.K.; ‡ Center of Medical Genetics, 26660 Universiteit Antwerpen, Prins Boudewijnlaan 43/6, BE 2659 Edegem, Belgium; § Groningen Biomolecular Sciences and Biotechnology, University of Groningen, Nijenborgh 7, 9747AG Groningen, The Netherlands

## Abstract

The emergence of fusion proteins that express the ROS1 kinase domain has become a promising target in non-small-cell lung cancer (NSCLC). Although earlier kinase inhibitors effectively managed ROS1-positive tumors, the rise of point mutations, particularly those beyond the binding pocket, has challenged the inhibitor efficacy. Notably, mutations at residue F2004, which cause cysteine or valine substitution, exhibit intriguing response profiles to the inhibitors. These mutations respond to small molecules that target the active conformation of the kinase (type I) but resist inhibitors that explore the inactive conformation (type II). Our study generates a ROS1 kinase model and uses molecular dynamics simulations to discern structural differentiators of the inactive conformation. A hydrophobic cluster within the active site, involving DFG residue F2103, demarcates the active conformation. We unveil insights from F2004C/V mutations in the ROS1 kinase domain from both the active and inactive states. Notably, the mutations do not perturb the active conformation, resembling wild-type (WT) ROS1. However, in the inactive conformation, the mutations disrupt the flexibility of DFG residue F2103, stabilizing the hydrophobic cluster. Our results provide a model for the inactive conformation of the elusive ROS1 kinase domain, offering pivotal insights into potential differences from the active conformation. Furthermore, our study of F2004C/V mutants proposes a plausible mechanism underlying the type I or II inhibitor response.

## Graphical abstract

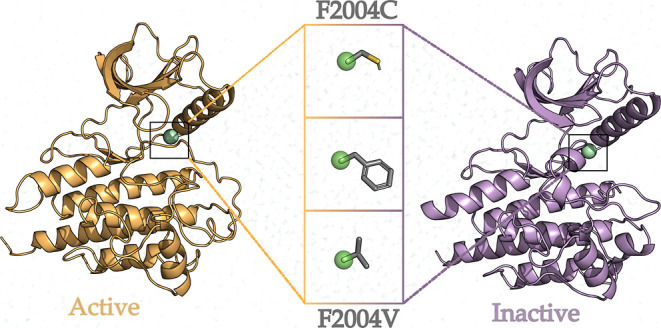

## Introduction

The crucial role of kinases in biological processes has been widely acknowledged. These proteins, with their transferase activity, activate downstream pathways by transferring phosphate groups to substrate proteins. Native kinases exhibit the ability to transition between active and inactive states.^1^ The inactive state of a kinase assumes a conformation that hinders ATP molecule binding, primarily due to the distinct conformations of conserved motifs (such as the DFG motif) and alterations in regulatory motifs (like the regulatory αC-helix and activation loop).^2^ While characterizing the inactive conformation of kinases is challenging, glimpses into a subset of specific kinases’ inactive conformations have laid the foundation for subsequent research.^3–5^ These insights into the inactive conformation of select kinases have acted as stepping stones for further investigations in this intricate area.

Characterizing the inactive structures of prominent kinases such as Src-kinase, epidermal growth factor receptor (EGFR), anaplastic lymphoma kinase (ALK), and others underscores the pivotal role of the aspartate–phenylalanine–glycine (DFG) motif in determining the kinase state.^5–7^ In the active conformation, phenylalanine within this motif is buried at the bottom of the catalytic αC-helix. At the same time, the aspartic acid side chain protrudes into the ATP-binding pocket. This configuration, termed DFG-in, facilitates the stabilization of the ATP phosphate group and magnesium ion through interactions with the aspartic acid side chain.^8^ Conversely, the DFG-out conformation is marked by the phenylalanine side chain of the DFG motif occupying the catalytic pocket, hindering ATP binding. Simultaneously, the rotation of the αC-helix and structural shifts in the activation loop collectively contribute to the stabilization of the inactive state.^9^

The determination of the inactive state of a kinase has proven to be instrumental in designing kinase inhibitors that effectively target this conformation. This strategy capitalizes on the specificity pocket, a cavity that emerges upon the rotation of DFG phenylalanine.^10^ These inhibitors are categorized as type II inhibitors, with prominent instances like imatinib and sorafenib finding widespread application in clinical kinase inhibition.^11,12^

Aberrant kinase activity stands as a hallmark of cancer, often attributed to oncogenic events encompassing gene amplification, activating mutations, and fusion proteins.^13^ A notable case involves the oncogenic fusion event where the ROS proto-oncogene 1 (ROS1) kinase domain fuses with various partners.^14^ Across diverse cancer types in both adults and children, ROS1 fusion proteins have been identified as significant drivers of oncogenesis. Intriguingly, akin to ALK, fusion occurrences involving ROS1 retain the core kinase domain.^14^ Moreover, a striking resemblance in the catalytic pocket between these two kinases has been observed, thereby enabling the repurposing of ALK inhibitors endorsed by regulatory bodies to target ROS1 fusions effectively.^15,16^

Despite the initial success achieved by repurposing type I ALK inhibitors, namely crizotinib, and lorlatinib, their efficacy was compromised by the emergence of mutations in the context of ROS1^+^ management.^17,18^ These mutations, predominantly found within the ATP-binding pocket, exert a disruptive influence on the interactions with these small-molecule inhibitors. Notably, mutations such as the gatekeeper mutation L2026 M and the solvent front G2032R mutation disrupt or entirely abrogate the binding affinity of crizotinib or lorlatinib to the ROS1 kinase domain.^15,19,20^

Interestingly, subsequent investigations revealed the potency of type II inhibitors, specifically cabozantinib, and foretinib, in addressing mutations, both within and outside of the binding pocket.^15,20,21^ Despite being positioned outside the confines of the binding pocket, certain mutations can indirectly foster drug resistance by engaging with critical regulatory motifs essential for drug binding. These encompass the glycine-rich loop, the activation loop housing the DFG motif, and the regulatory αC helix. The regulatory helix and the activation loop establish a hydrophobic interaction interface, involving key residues including F2004 at the base of the helix and phenylalanine 2103 from the DFG motif within the activation loop.^18^ In the active conformation of the kinase, the DFG motif exposes the side chain of D2002 toward the ATP-binding pocket while simultaneously embedding the aromatic side chain of F2103 toward the terminus of the helix.

Earlier studies, informed by the structural characteristics of inactive kinases and employing a model of the ROS1 kinase domain based on an inactive ALK structure, introduced the concept of a rotational shift in the DFG motif. This shift leads to the exposure of the side chain of F2103 to the ATP-binding pocket, consequently forming a new pocket in the crevice between the helix and the loop. The side chains of F2004 and F2075 demarcate this pocket. Upon binding of cabozantinib and foretinib, this pocket is effectively occupied by the shared fluorophenyl ring present in both drugs.^15,21^

Mutations in the F2004 residue have been correlated to a reduction in the binding affinity of type II drugs. Whether it involves a substitution to cysteine or valine, this mutation can directly impede the stabilization of the fluorophenyl ring, leading to observable increases in the IC_50_ values within ROS1^F2004C/V^ mutant-bearing Ba/F3 cells.^15,20^ The use of computational tools, especially molecular dynamics simulations, provides an advantageous insight into the structural aspects of protein dynamics and possible mutations at an atomistic level.^22–25^ To elucidate the mechanisms underpinning the resistance of the mutations F2004C and F2004 V in the ROS1 kinase domain, the current study carried out molecular dynamics simulations of the ROS1 kinase domain in both active and inactive conformations. These investigations provide valuable insights into the intricacies of resistance engendered by these mutations.

## Results

Despite solid reports on the active–inactive structure of various kinases, to date, no experimentally determined structure of the ROS1 kinase domain has been reported in its inactive conformation.^15,26^ In this scenario, the use of homology modeling can cover this gap using the structure of the inactive conformation of a homologous kinase as a template to generate a model of an inactive ROS1 kinase domain.

Within the kinome, ALK shows a high homology to ROS1, especially in the catalytic pocket. In addition, there are a multitude of structures for ALK, including the inactive conformation. For the selection of a suitable ALK structure, our main filter was the presence of the “DFG-out” and, in a second stance, the presence of a short helix at the N-terminal end of the activation loop. The DFG-out conformation is represented by the side chain of aspartate, part of the DFG motif, being positioned toward the bottom of the regulatory αC-helix, while the side chain of the phenylalanine is positioned toward the active site.^10^

From the available structures, the crystal structures deposited under the PDB IDs 4FNY and 3L9P were selected. Given the advance of AI tools such as ChaiDiscovery, we also submitted the ROS1 kinase domain in the presence or absence of cabozantinib, a type II inhibitor, aiming to obtain a plausible model of the inactive conformation of ROS1.^27^ The ALK PDB 3L9P was discarded because despite presenting a short helix in the N-terminal end of the activation loop, the DFG phenylalanine was buried at the end of the regulatory αC-helix, characterizing an active DFG-in state (Figure 1).

**Figure 1:**
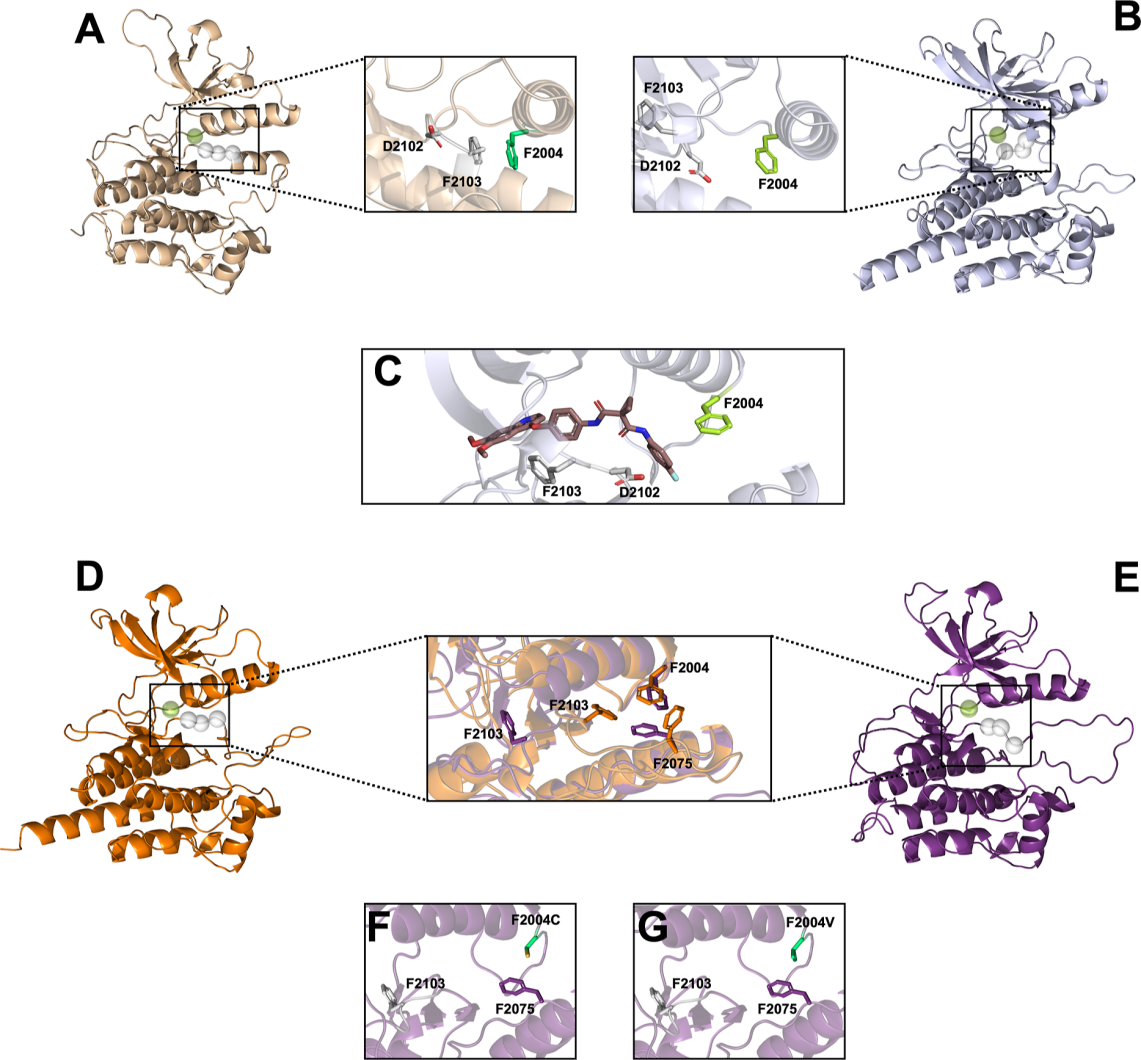
Structural representation of the ROS1 kinase domain and mutational impact using different templates for modeling. Depiction of ROS1 in the kinase domain obtained using the template (A) PDB 3L9P and the (B) ChaiDiscovery server, with a highlight in the area containing residues F2004, D2102, and F2103. (C) Representation of the ligand cabozantinib in the ROS2 kinase binding site, highlighting the interactions with residues F2004 and F2103. (D) The active conformation of ROS1 used in this study was obtained from the optimization of PDB 3ZBF, while the (E) inactive model was obtained with homology modeling using the template PDB 4FNY. The highlighted area shows the superimposition of the active crystal structure (PDB ID: 3ZBF) and the modeled inactive state of the ROS1 wild-type kinase domain, highlighting the hydrophobic stacking interactions involving residues F2004, F2075, and F2103. This interaction is illustrated for the (F) F2004C variant and the (G) F2004 V variant.

Our models obtained from ChaiDiscovery from submitting only the ROS1 kinase domain sequence (Supporting Information Table S1) yielded only the active conformation. However, by adding the SMILES of the ligand cabozantinib in the pipeline (Supporting Information Table S1), we were able to obtain a model of the ROS1 kinase domain in an inactive state in complex with cabozantinib in a pose resembling previous descriptions (Figure 1 and Supporting Information Figure S1).^15^ After the ligand was extracted, the apo system was simulated, as described in Experimental Section. After the analysis of the RMSD and the rotation of DFG phenylalanine (Supporting Information Figure S1), it was observed that this model resembles the results obtained from simulating our homology model using the PDB 4FNY template (Supporting Information Figures S2–S5). Given that template 4FNY was experimentally determined with adequate resolution (2.45 A), this structure was selected to follow-up with our studies. In this paper, the optimized 4FNY structure was used as a template to model the three inactive structures in the apo form: (i) wild type (WT) and mutants (ii) F2004C and (iii) F2004 V (Figure 1). All simulations were stable after the equilibration time (Supporting Information Figures S2–S5), and the equilibrated trajectories of 18 × 0.6 μs in total (WT and both mutants, in both active and inactive conformations, three replicas each) were analyzed.

### Different Networks of Interactions Stabilize the Active and Inactive Conformations

Changes in the network of interaction between the active and the inactive state in other kinases have been previously described but not yet fully for ROS1.^28–30^ Our analysis focused on all possible salt bridges within the kinase domain. This type of interaction contributes to protein stabilization and might also contribute to the binding of substrates. In kinases, a specific salt bridge connecting the β3-strand and the αC-helix is pivotal for ATP binding. In our analysis, it is possible to label two classes of salt bridges: (a) majorly present in the active trajectories and (b) majorly present in the inactive trajectories. The active trajectories for the WT salt bridges involving the pair of residues, K1980/E1997, K1983/E1990, K1991/D2020, E1993/K1996, and D2058/R2219, are consistent and interestingly less present in the WT inactive simulations (Figure 2). In parallel, the inactive trajectories for the WT salt bridges involving residues E1961/K1976, K2011/E2027, K1980-D2102, K1991/D2020, E1958/K1983, E2131/R2205, E1990/K2111, and D2135/K2117 show a higher occupancy than that observed in the active trajectories.

**Figure 2:**
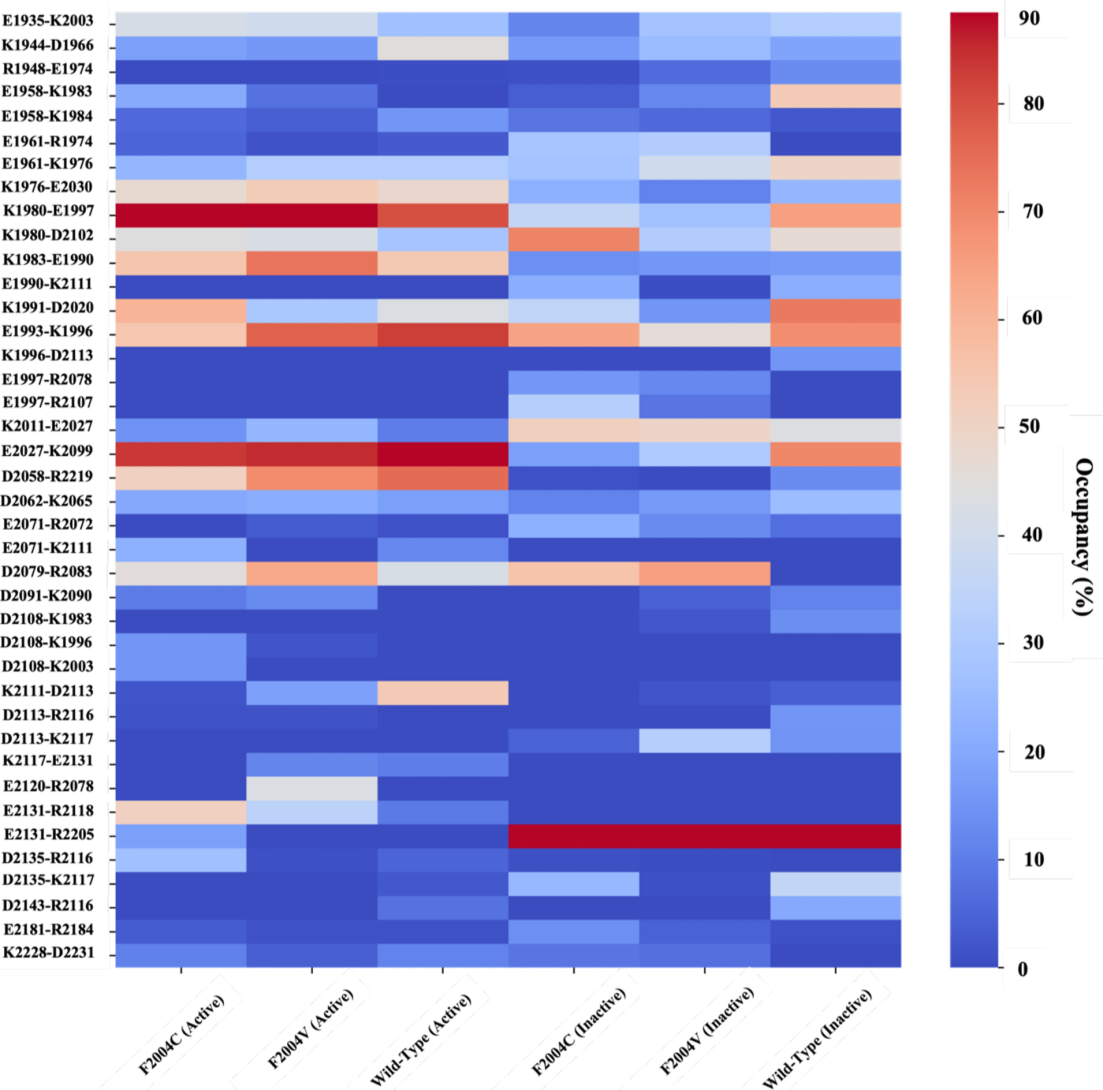
Salt bridge interaction network analysis. Trajectories were scrutinized for potential salt bridge interactions using a 0.4 nm cutoff (heavy atom to heavy atom), and their presence across frames was illustrated in a heatmap. For each variant, replicate trajectories were concatenated, and the combined occupancy was computed. The heatmap displays salt bridges present in at least one variant, with the occupancy exceeding 15%, represented by a color gradient ranging from dark blue (lower occupancy) to dark red (higher occupancy).

From Figure 2, it is possible to identify a trend previously described in other kinase domains.^31^ The K1980 (β3-strand)–E1997­(αC-helix) salt bridge is a hallmark of the active state due to its crucial role in stabilizing any ATP molecule anchored to the active site. This interaction is decreased in the inactive state due to a probable reorganization of the activation loop leading to the rotation of the DFG from an in into an out state. This rotation not only hampers the K1980–E1997 interaction but also favors the interaction of K1980 and D2102 in the inactive conformation. This trend is also observed in the trajectories of the mutated proteins; the active trajectories of mutants F2004C or F2004 V display a high occupancy of K1980–E1997, while the inactive ones show a lower value for this salt bridge but favor the K1980–D2102 salt bridge.

The composition of the salt bridges in the active simulations is mostly linked to the stabilization of the N-terminal lobe. The exception is observed for interactions D2079/R2083, involving the aspartate from the HRD motif, and D2058/R2219. The HRD motif is, together with the DFG motif, part of the central hub of interactions of the kinase domain and consequently plays a pivotal role in the signaling network due to its role as a stabilizing component of the active conformation. The salt bridge maintains the correct positioning of the HRD and consequently of the R-spine, a three-dimensional organization of residues of the kinase domain that ensures proper activation of the enzyme. As expected, D2079/R2083 showed low occupancy in the WT simulations; however, it is remarkable to notice that in both mutants the occupancy of this interaction is comparable to that of the WT, indicating a different arrangement of the activation loop from the inactive WT trajectories. A few interactions in the active simulations are shown to be more prevalent in both mutants when compared with the WT, such as R2107–I2076 and R2078–D2143, regions in the neighboring region of relevant residues 2075 and the DFG motif.

The correlation of the HRD and DFG motifs also involves hydrogen bonds (H-bonds). In this case, we screened all possible H-bonds during the simulations involving the residues of the DFG or HRD motifs (Figure 3). In this search, it was possible to identify H-bonds involving the H2077 and DFG-1 residue (G2101), R2078, and the DFG+1 (L2105) residue, in the active WT simulation, which were absent in the inactive counter-partner. Additionally, the HRD motif is also anchored to the highly stable αF-helix through H-bonds with the residue D2143. Likewise, as observed for the salt bridge analysis, these interactions are reduced in the inactive WT simulations. On the other hand, interactions of the HRD specific to the inactive WT trajectories were scarce with only one minor example, R2078–D2102 (Figure 3).

**Figure 3:**
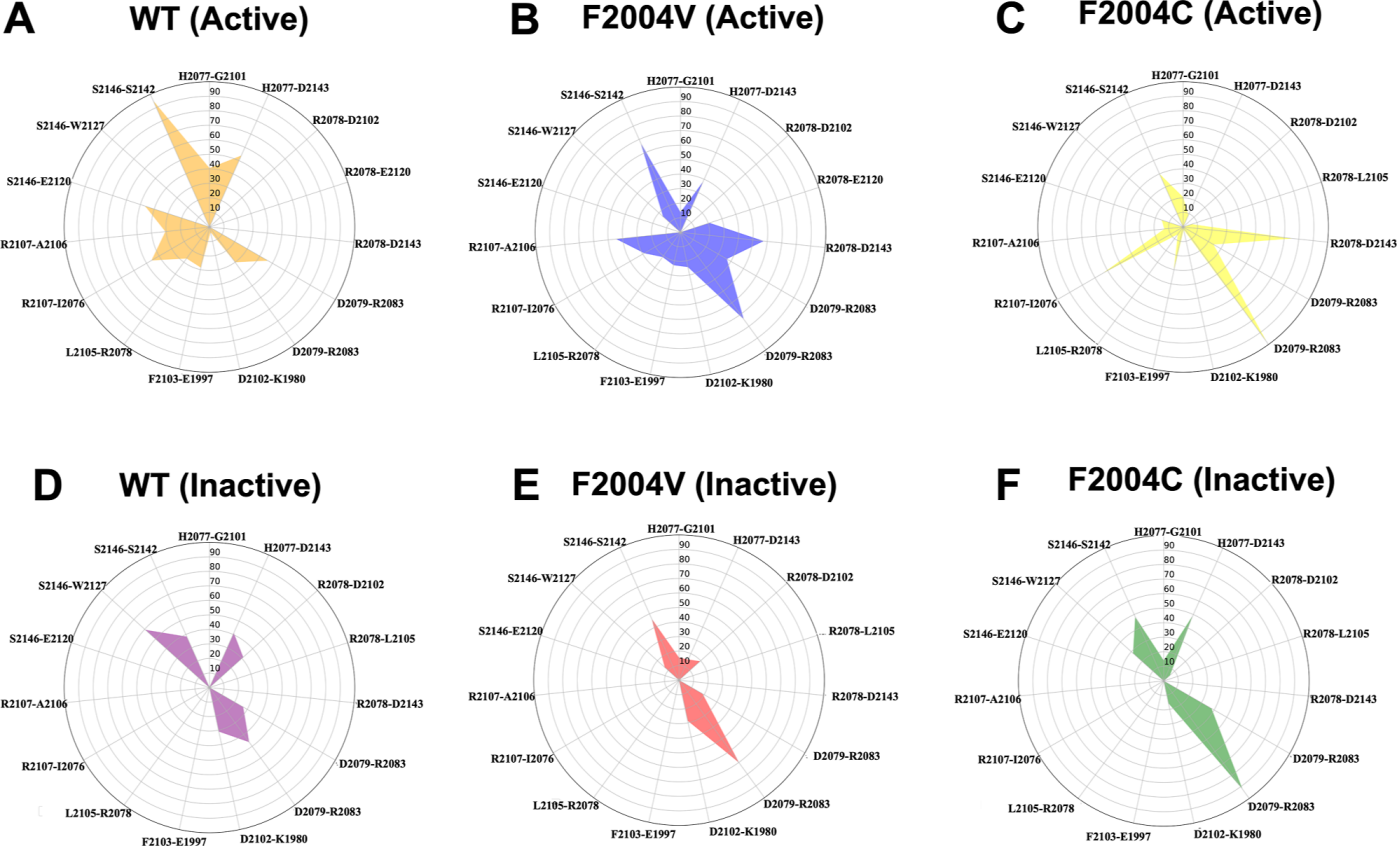
Radar plot illustrating interaction network dynamics. This radar plot showcases the occupancy percentages (%) of hydrogen bonds within different ROS1 kinase domain variants: wild-type in its active (A) and inactive (D) states, F2004 V mutant in the active (B) and inactive (E) states, and F2004C mutant in the active (C) and inactive (F) states. The occupancy values represent the proportion of frames observed across all triplicates where the specified hydrogen bonds were detected.

When analyzing the same network of interactions for the mutants, the trend was less well-defined. Concerning the active simulations of mutants F2004C and F2004 V, a steep decrease was detected in the H2077–G2101 and R2078–L2105 H-bonds. Meanwhile, the aspartate from HRD engages in a steady H-bond with R2083 in both mutants. Interestingly, this interaction is also highly stable in the inactive simulations of both mutants (Figure 3). When comparing the inactive simulations, it is possible to identify a comparable profile between mutants, with an increased occupancy of bond D2079–R2083 and a decrease in bond occupancy between residues S2146 and S2142. Despite our success in differentiating the active and inactive states of ROS1^WT^, more insight into the effect of the mutations is needed, aiming to understand its response to the available therapy or even for the development of specific inhibitors.

### The F2004C/V Mutations Impact the Pocket Volume by Restraining the Rotation of the DFG Motif in the Inactive Conformation

The DFG state also controls the presence of a hydrophobic cluster at the bottom of the regulatory αC-helix. In the DFG-in state, the side chain of the F2103 residue is buried close to the bottom of the αC-helix while engaging in hydrophobic stacking interactions with F2004 and F2075 (Figure 1). As such, the rotation of the DFG motif can be used to differentiate between the active and inactive conformations by determining which residue of the DFG motif occupies the catalytic pocket. Consequently, the state of the DFG can directly impact the pocket volume; the rotation of the DFG motif exposes the aromatic side chain of the phenylalanine to the catalytic site in the DFG-out state. In other kinases, the phenylalanine aromatic ring engages in a network of hydrophobic interactions with the N-terminal β-sheets and αC-helix that stabilize the inactive conformation.^5,7^

An angle determined by the α carbon of residues E1980 (β4-strand), K1997 (αC-helix), and F2103 (DFG) was used to characterize the DFG state; this angle allowed us to calculate the rotation of the DFG motif based on the positioning of the phenylalanine. Our simulations of the active WT ROS1 kinase domain displayed a steady DFG-in conformation, indicating a stable active conformation (Figure 4) despite a bimodal distribution of the DFG rotation angle. The bimodal distribution observed for the active WT simulation can be associated with small fluctuations of the β-sheet (Supporting Information Figure S4). Additionally, the DFG phenylalanine side chain remained buried at the bottom of the αC-helix. In parallel, the same calculations were performed for the simulations of the inactive ROS1^WT^ kinase domain, and it was possible to see a clear distinction in the conformation of the DFG motif between both conformations (Figure 4).

**Figure 4:**
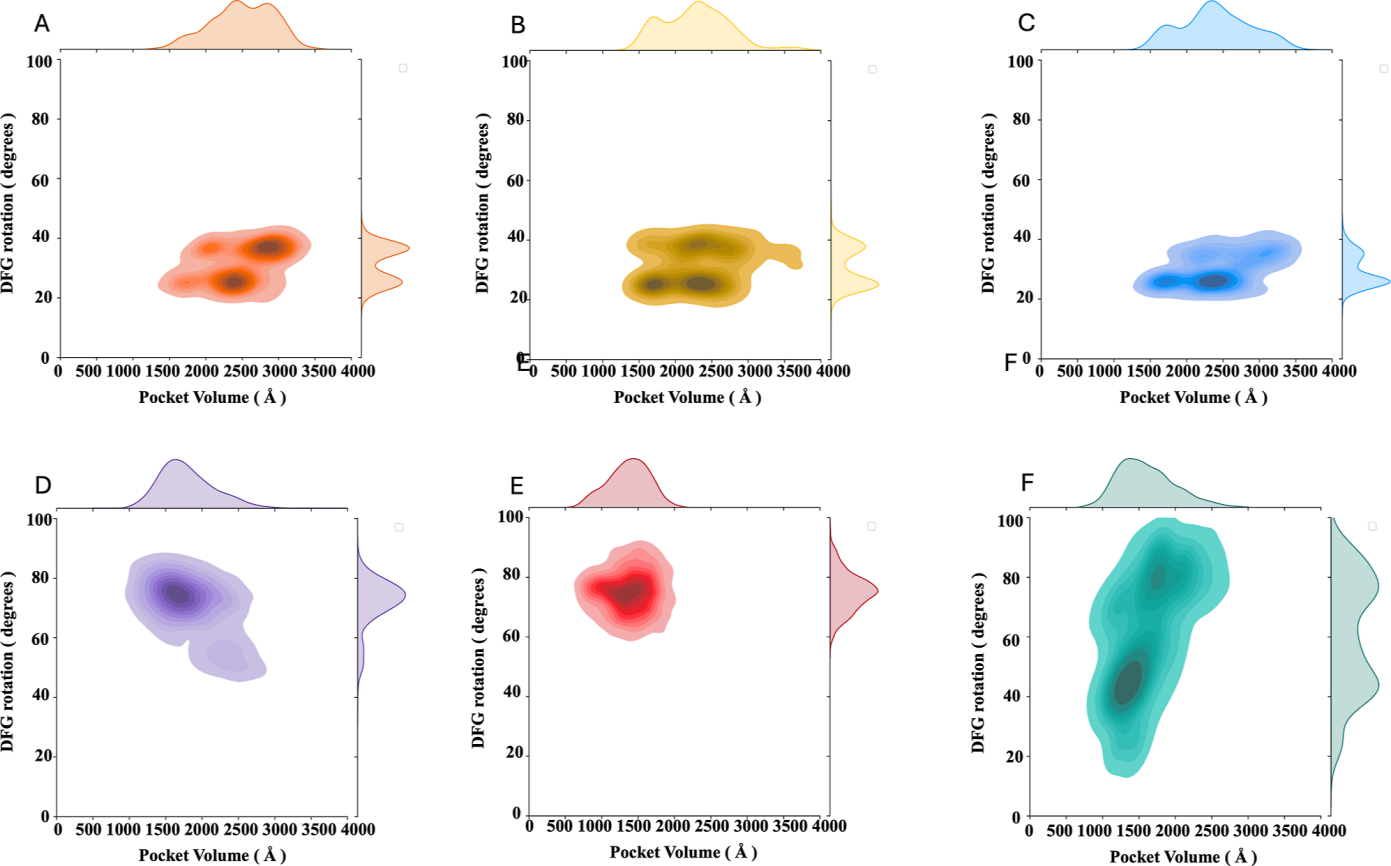
Correlation between DFG rotation and ATP-binding pocket volume. This joint plot illustrates the correlation between DFG rotation, measured as an angle based on the α carbon of residues K1980–E1997–F2103, and the ATP pocket volume across active and inactive simulations for different ROS1 kinase domain variants: (A/D) wild-type, (B/E) F2004C, and (C/F) F2004 V. Each variant’s distribution histogram for both variables is also independently presented along their respective axes.

In the WT inactive plot, the major peak (75°) corresponds to the side chain of DFG phenylalanine fully occupying the ATP-binding pocket. In this conformation, the hydrophobic aromatic side chain is stabilized by interaction from a hydrophobic network involving residues from the G-loop (L1951), β2 strand (V1959), β3 strand (A1978), β4-α loop (L2010), hinge (L2026), and β6 strand (L2086) (Figure 5). This network stabilized the aromatic side chain and consequently the inactive state. The broader region represents a different conformation of the DFG motif; although the phenylalanine side chain is not buried close to the end of the αC-helix, it is pointed toward the C-terminal of the activation loop. This conformation also correlates with a larger pocket volume (Figure 4).

**Figure 5:**
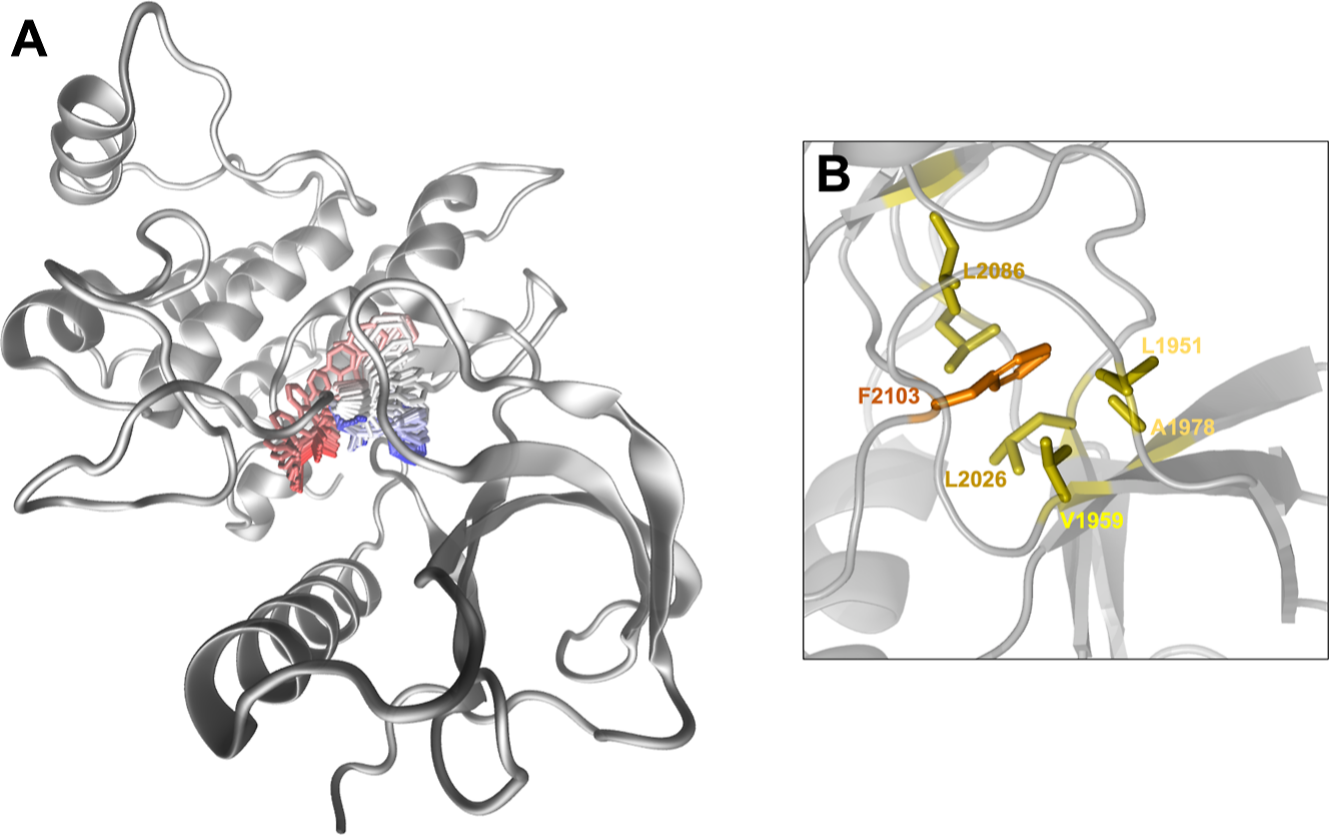
Stabilization of DFG rotation in inactive simulations through hydrophobic interactions with the ATP-binding pocket. (A) Depiction of the inactive ROS1 kinase domain’s backbone in gray, accompanied by a representation of the trajectory of the F2103 side chain in the wild-type simulation. Atom colors transition from red (beginning of the trajectory) to blue (end). (B) Highlights of the hydrophobic cluster identified during wild-type simulations, where the F2103 side chain occupies the ATP-binding pocket and engages in van der Waals interactions with L1951, V1959, L1978, L2026, L2080, and L2026.

It is interesting to highlight that certain conformations achieved by the DFG motif in the inactive simulations direct the side chain of phenylalanine toward the αC-helix; however, this conformation does not seem to be stable and quickly returns to a more conventional DFG-out conformation (Figure 5).

Considering the ROS1^WT^ kinase domain, the average angle found for the active simulations of 31° ± 6° (SD) is notably smaller than the average angle found for the inactive simulations of 72° ± 8 (SD). The pocket volume calculation also showed a striking difference between the active and inactive simulations; while for the WT active simulations, this pocket averaged 2.5 ± 0.4 nm^3^ (SD), the inactive simulations averaged 1.8 ± 0.4 nm^3^ (SD) (Figure 4).

Due to the successful classification of conformations based on the correlation of the DFG rotation and the pocket volume for WT simulation, we extended this analysis to the mutants. From Figures 2–4, it is possible to imply that mutations of the phenylalanine into either a cysteine or a valine lead to major conformational changes that could lead to a change from an active to an inactive conformation. Thus, the variability observed can be associated with the intrinsic disorder associated with the active conformation of kinase domains. The simulations of the mutants starting from the active conformation strike a high similarity to the profile observed for the WT active simulation; there is a conservation of the network of interaction (Figures 2 and 3), and the DFG-in conformation and the pocket volume are also highly comparable (Figure 4).

Concerning the inactive simulations of the mutants, a few aspects are comparable. Both mutants display a decrease in the K1980–E1997 salt bridge occupancy, as would be expected for a kinase in its inactive conformation (Figure 2). In addition, the network of interactions maintaining the HRD motif in place and consequentially the R spine is also absent. However, there are important differences not only in comparing the mutants with each other but also with the WT. Mutation F2004C leads to a more constrained DFG motif, with the F2103 side chain engaging in a hydrophobic cluster with pocket residues, as observed for the WT simulations. However, this changes once the phenylalanine is mutated into a valine; in addition to a noteworthy decrease in the pocket size, the DFG rotation angle shows the biggest variation. In the F2004 V mutant, the pocket achieves the lowest volume. Analysis of the trajectories associates this volume with a reorganization of the glycine-rich and activation loops, with the latter folding on top of the former (Supporting Information Figure S6). However, it is important to highlight that neither in this conformation nor any other observed for the F2004 V mutant does the DFG motif rotate enough to display a DFG-in conformation; the same can be stated for the F2004C inactive trajectories.

## Discussion and Conclusion

Kinases exhibit remarkable dynamism, which is a trait critical for precise cellular regulation. Interestingly, the same structural changes that facilitate ATP binding in the active conformation can impede this process in the inactive state.^32^ Moreover, point mutations within the ROS1 kinase domain have been associated with oncogenic advantages and resistance to clinically approved drugs. While mutations within the ATP-binding pocket have received extensive scrutiny, newly emerging mutations within neighboring motifs around the catalytic pocket demand further investigation.^15,18,26^ As observed with other mutated kinases, understanding the mechanisms behind catalytic activation or drug resistance is essential. This understanding not only aids in selecting the most effective therapeutic strategies but also contributes to the development of innovative selective inhibitors.^33–36^

In this study, our initial challenge revolved around the absence of an experimentally determined structure for the ROS1 kinase domain in the inactive conformation. The absence of an experimentally determined structure for the inactive conformation of the ROS1 kinase poses a significant obstacle to understanding the dynamics of this kinase and developing type II inhibitors tailored specifically for ROS1, which could significantly benefit cancer patients. We addressed this bottleneck by employing homology modeling, using as a template the X-ray crystal structure (PDB ID: 4FNY) of a related kinase known as ALK, as previously reported by Davare et al. and others.^6,15,20^ Utilizing this model of the wild-type (WT) inactive ROS1 kinase domain as our foundation, we embarked on a dual approach, conducting molecular dynamics simulations for both the inactive and active conformations of the same protein.

Our research successfully reproduced a stable active conformation, consistently preserving the DFG-in state across three independent simulations of the active conformation. Additionally, our analysis unveiled a network of interactions within the active simulations that had been previously documented, which includes the enduring K1980–E1997 salt bridge and a hydrogen-bond network connecting the HRD motif to the DFG loop.

In our simulations of the WT inactive conformation, the initial structure in all simulations displayed a DFG-out state, with the phenylalanine side chain exposed and the aspartate directed toward the regulatory αC-helix. However, our analysis of the simulations revealed a substantial degree of flexibility in the DFG motif, particularly in the phenylalanine residue. Despite the rotational movements allowing the aromatic ring to approach the αC-helix, it subsequently reverts to the catalytic site, forming a hydrophobic cluster in conjunction with residues L1951, V1959, A1978, L2010, L2026, and L2086. The presence of such a hydrophobic cluster in inactive kinases has been previously observed in other kinase structures and is believed to contribute significantly to stabilizing the inactive conformation.

The occurrence of mutations is a common phenomenon in kinase-driven tumors, primarily driven by the selective pressure exerted by the use of kinase inhibitors. Within the ROS1 kinase, a multitude of point mutations have been documented. Among these mutations, those occurring at position 2004 are of particular interest due to their distinct responses to different types of inhibitors. These mutations display sensitivity to type I inhibitors, which target the active conformation but exhibit reduced efficacy when faced with type II inhibitors designed for the inactive conformation. Furthermore, it is worth noting that in all available crystal structures of ROS1, residue F2004 is involved in hydrophobic stacking interactions with both F2103, a component of the DFG motif, and F2075.

In our initial analysis of independent simulations for mutants F2004C and F2004 V in the active conformation, we observed a conservation of the active state, with pocket volumes closely resembling those of the wild-type (WT) variant. However, intriguing variations emerged when simulating these mutants in the inactive conformation. Notably, neither of the mutants exhibited the characteristic “swing” motion for the DFG motif observed in the WT kinase domain in the inactive simulations, which is associated with the rotation of the phenylalanine side chain. In the case of F2004C simulations, we observed a highly stable conformation of the DFG motif with the phenylalanine forming a hydrophobic cluster, mirroring the behavior of the WT domain. Conversely, in the F2004 V simulations, an alternative state emerged, while the DFG motif also adopted a conformer with the hydrophobic cluster. In this state, the activation loop pushed the glycine-rich loop toward the active site, resulting in the smallest pocket size observed in our study.

Based on our initial findings, it can be inferred that both mutants do not significantly disrupt the delicate equilibrium between the active and inactive conformations achievable by the kinase domain. Furthermore, the mutants’ response to type I inhibitors can be elucidated by the remarkably stable active conformation maintained within the kinase domain, even in the presence of these specific point mutations. These conformations closely mirror the behavior of the WT, providing a conserved active pocket suitable for binding both ATP and type I inhibitors.

The intriguing plasticity exhibited by this is worth noting; despite most WT inactive simulations suggesting a state where F2103 occupies the binding pocket, the inherent flexibility of the protein allows for the rotation of the aromatic ring, enabling the binding of small molecules like type II inhibitors, as documented in the literature. In parallel, the inability of mutants F2004 V and F2004C to attain similar conformations, resulting in either an exceedingly compact pocket or the persistent presence of the aromatic ring from the DFG motif, can be postulated as a hypothesis concerning the mechanism behind the resistance of these mutants to type II inhibitors. However, it is imperative to emphasize that further investigations, particularly involving an in-depth analysis of receptor–ligand interactions, are warranted to substantiate this hypothesis.

## Experimental Section

### Protein Preparation

The three-dimensional crystal structure for the active ROS1 kinase domain which served as a template for modeling ROS1 structures, obtained from the Protein Data Bank (PDB, ID: 3ZBF), showed an X-ray resolution of 2.2 Å.^18^ The initial PDB structure was stripped of bound ligands, cofactors, and water molecules. These structure refinements were performed using UCSF Chimera.^37^ Missing residues were modeled using Modeler. To model the selected mutants, UCSF Chimera was used by exploiting the “Rotamers” feature. Position 2004 was mutated, and the desired rotamer was selected. For side chain rotamers’ selection, the ones with the highest probability were selected.^38^ These models were saved independently for each mutant and used for further studies.

For the inactive structure of ROS1, our initial search yielded PDB IDs 4FNY and 3L9P. Models were obtained through the use of the “homology modeling” tool available at the Chimera software. Chai Discovery is an AI web server for studying protein folding either in the presence or absence of ligands. The ROS1 sequence and ligand SMILES submitted are presented in Supporting Information Table S1. The selected inactive ALK crystal structure (PDB ID: 4FNY) was used as a template for the final inactive ROS1 kinase domain. The initial PDB structure was stripped of bounded ligands, cofactors, and water molecules. Upon aligning with the ROSWT sequence, the “homology modeling” feature of Modeler within the UCSF chimera software was used to generate an ensemble of models of the inactive ROS1 kinase domain.^38^ The ensembles’ quality was assessed using the discrete optimized protein energy parameter, with the one with the highest score being considered the highest quality and being further selected for molecular dynamics simulations.

### Molecular Dynamics Simulation Setup

Apo-ROS1 models, referring to the protein without cofactors, were then subjected to MD simulation. For initial system preparations, GROMACS (Version 2021.3) was used, and production runs were carried out using Version 2021.1,d, depending on the version available in the HPC clusters.^39^ CHARMM36 force field (version 2020) was applied to the systems, and TIP3P water was used for solvation with a solute–box distance of 10 Å in a dodecahedral box.^40^ Counterions Na^+^/Cl^–^ were used to neutralize the system and achieve a 150 mM concentration before performing energy minimization. Steepest descent minimization was used for energy minimization with a maximum of 50,000 steps. A two-step equilibration was performed starting with a canonical ensemble under a constant number of particles, volume, and temperature (*NVT*) for 200 ps and an *NPT* ensemble with constant number of particles, pressure, and temperature. LINCS constraints were applied on bonds involving hydrogens, and the Verlet scheme was used for nonbonded settings using a cutoff of 10 Å for both short-range electrostatics and van der Waals. Particle mesh Ewald for long-range electrostatics was employed.^41,42^ Temperature coupling was performed with a modified Berendsen thermostat (V-rescale) at 300 K (tau_t = 0.1) and a Parrinello–Rahman barostat (tau_p = 2).^43,44^

### Analysis Details

After successful production runs, structural and conformational analyses were applied using the GROMACS toolkit. Important interactions between the residues of potential relevance were listed after the visual inspection of the initial structure; hydrogen-bonding and salt bridge analyses, including distance and angle measurements, were performed to study the system evolution and stability of these interactions. For visual inspection of structural features and trajectory analysis, visual molecular dynamics and PyMol were utilized.^45^

Each simulation trajectory was studied by using an ensemble of tools within the GROMACS package. Protein compactness and flexibility were explored using root-mean-square deviation (RMSD) and root-mean-square fluctuations (RMSF), respectively. Important interactions between the residues of potential relevance were listed after visual inspection of the initial structure; hydrogen-bonding and salt bridge analysis including distance and angle measurements were performed to study the system evolution and stability of these interactions.

To analyze the volume of the ATP-binding site of the ROS1 kinase domain, the simulations for each variant were concatenated, and the pocket volume was calculated every 1 ns. For the pocket calculation, a 1.5 × 1.5 × 1.5 nm^3^ cubic box was centered at the residue L2026 Cα atom. The defined box was filled with grid points at 0.1 nm resolution. Grid points that overlapped with any protein atoms were deleted. These steps were carried out using the program POVME3.^46^

## Supplementary Material



## Data Availability

Data accessibility: 10.5281/zenodo.15236275.

## Notes

Additional information on molecular dynamic simulations setup and analysis, including supporting figures (PDF)
