# Supplementary material for: Unveiling the Mechanistic Impact of Mutations F2004C/V in the ROS1 Kinase Domain
Source: ACS Omega. 2025 May 30;10(22):22837–46. doi: 10.1021/acsomega.5c00072 (PMC12163786; doi:10.1021/acsomega.5c00072)
Supplement: Supplementary file 1 [file ao5c00072_si_001.pdf]

Unveiling the mechanistic impact of mutations F2004C/V in the ROS1 kinase domain

Juliana F. Vilachã[*,†] , Farhan Ul-Haq[‡], Geert Vandeweyer[‡], and Siewert-Jan Marrink[¶]
[†] University of Warwick - Coventry Campus, School of Life Sciences Department of Chemistry, Gibbet Hill Campus, CV4 7AL, Coventry United Kingdom Coventry, UK CV47AL
[‡] Universiteit Antwerpen, Center of Medical Genetics, Prins Boudewijnlaan 43/6, BE2659, Edegem, Belgium
[¶]University of Groningen, Groningen Biomolecular Sciences and Biotechnology, Nijenborgh 7, 9747AG Groningen, The Netherlands
Author to whom correspondence should be addressed: juliana.vilacha@warwick.ac.uk

Supplementary Figures and Tables

Supplementary Figure 1. Details from simulations of the inactive ROS1 kinase domain obtained from the Chai Discovery web server.
Supplementary Figure 2. Root Mean Square Deviation calculation of the active ROS1 WT, F2004C, and F2004V simulations
Supplementary Figure 3. Root Mean Square Deviation calculation of the inactive ROS1 WT, F2004C, and F2004V simulations.
Supplementary Figure 4. Root Mean Square Fluctuation (RMSF) calculation of the **active** ROS1 WT, F2004C, and F2004V simulations.
Supplementary Figure 5. Root Mean Square Fluctuation (RMSF) calculation of the **inactive** ROS1 WT, F2004C, and F2004V simulations.
Supplementary Figure 6. Snapshot of the kinase domain with the G-loop highlighted in red and the side chain of the residue F2103 in orange.
Supplementary Table 1. Sequence and smile used for Chai Discovery models
Supplementary MDP files. Files for Energy Minimization, NVT and NPT ensemble and production simulation

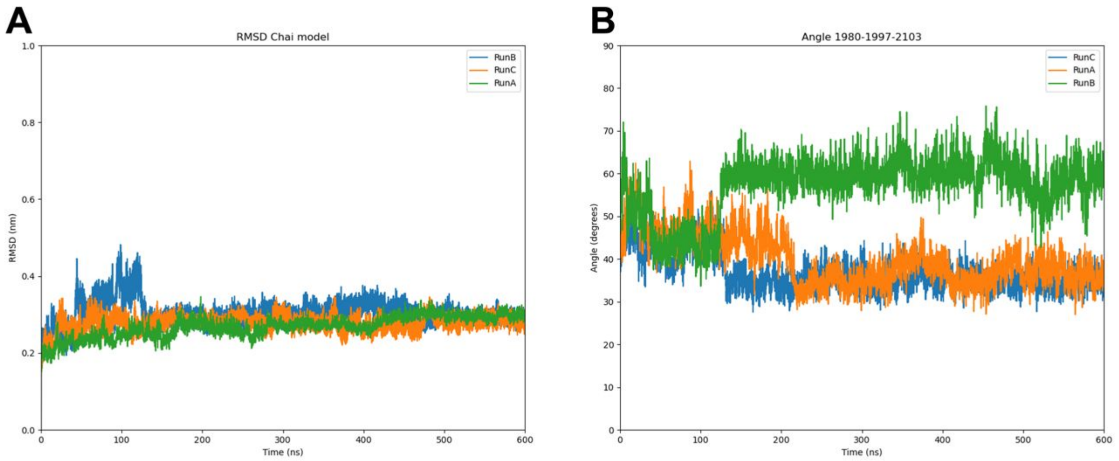

Supplementary Figure 1. Details from simulations of the inactive ROS1 kinase domain obtained from the ChaiDiscovery web server. (A)Root mean square deviation (RMSD). The RMSD was calculated by fitting the trajectory to the initial structure using backbone atoms. The RMSD were computed using 600 ns of MD simulation using three seeds. (B) DFG rotation. The rotation of the DFG motif was computed using an angle determined by the αC of residues 1980-1997-2103

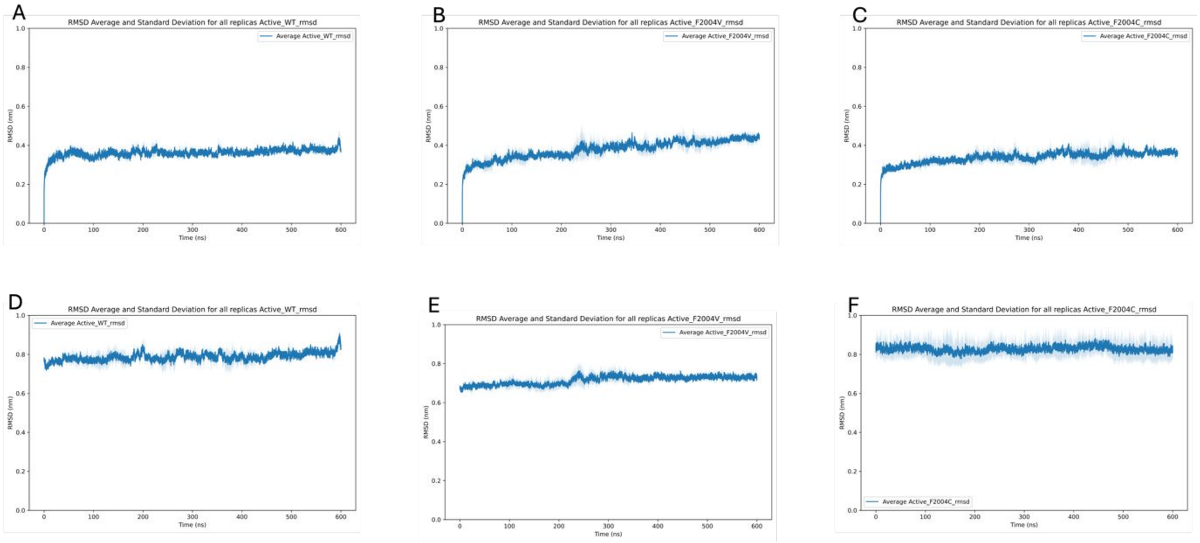

Supplementary Figure 2. Root Mean Square Deviation calculation of the active ROS1 WT, F2004C and F2004V simulations. The RMSD were computed using 600 ns of MD simulation using three seeds. Figures (A), (B) and (C) show the RMSD were calculated using the minimized and equilibrated structure of the active ROS1 kinae domain of the respective simulations. Figures (D), (E) and (F) show the RMSD were calculated using the structure of the **inactive** ROS1 kinase domain of the respective simulations

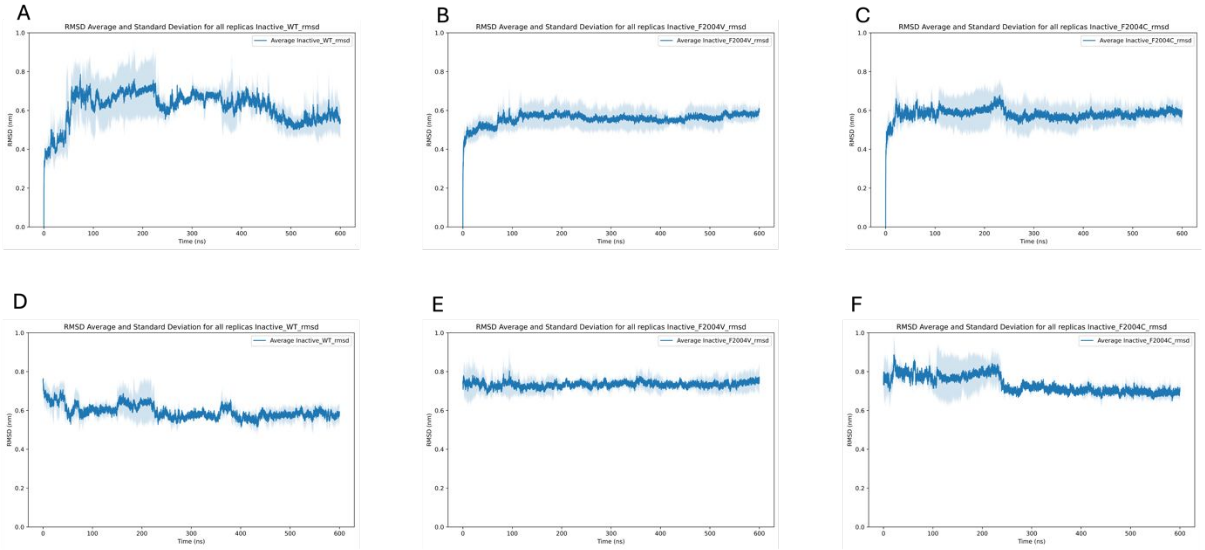

Supplementary Figure 3. Root Mean Square Deviation calculation of the inactive ROS1 WT, F2004C and F2004V simulations. The RMSD were computed using 600 ns of MD simulation using three seeds. Figures (A), (B) and (C) show the RMSD were calculated using the minimized and equilibrated structure of the inactive ROS1 kinase domain of the respective simulations. Figures (D), (E) and (F) show the RMSD were calculated using the structure of the **active** ROS1 kinase domain of the respective simulations

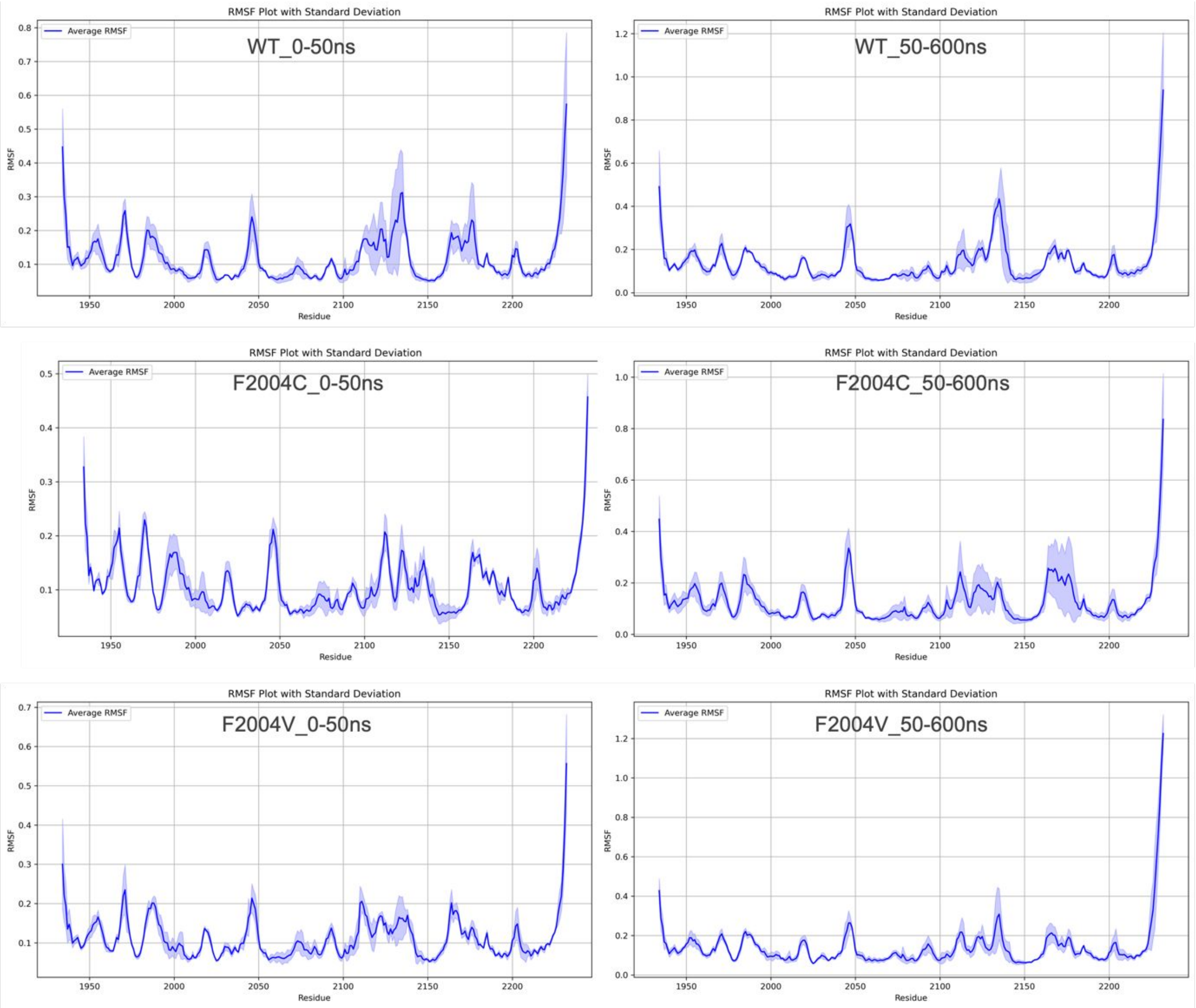

Supplementary Figure 4. Root Mean Square Fluctuation (RMSF) calculation of the **active** ROS1 WT, F2004C and F2004V simulations. The calculations were made for the protein based on the deviation of alpha carbon of the main chain of every residue. RMSF was calculated for the first 50 nanoseconds or last 550 nanoseconds, averaged and plotted with the standard deviation.

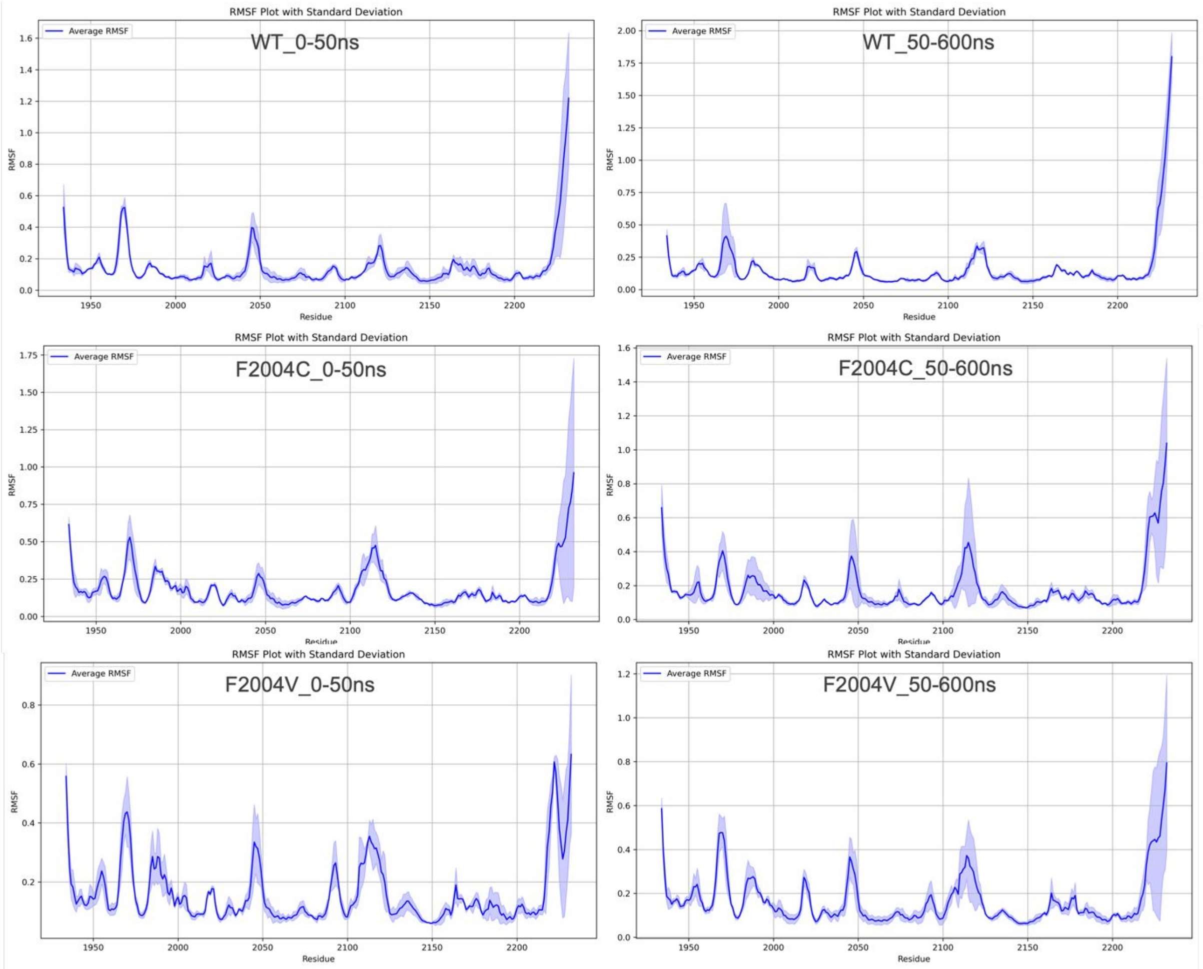

Supplementary Figure 5. Root Mean Square Fluctuation (RMSF) calculation of the **inactive** ROS1 WT, F2004C and F2004V simulations. The calculations were made for the protein based on the deviation of alpha carbon of the mainchain of every residue. RMSF was calculated for the first 50 nanoseconds or last 550 nanoseconds, averaged and plotted with the standard deviation.

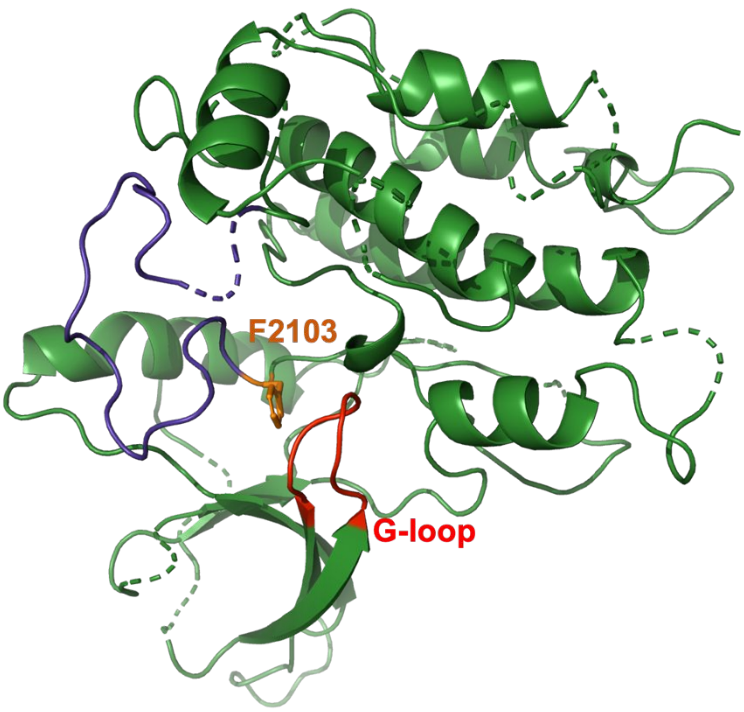

Supplementary Figure 6. Snapshot of the kinase domain with the G-loop highlighted in red and the side chain of the residue F2103 in orange. This snapshot represents the conformation of the phenylalanine 2103 located on top of the G-loop

Supplementary Table 1.Sequence used for Chai Discovery models

ROS1 sequence	Cabozantinib (SMILES)
IENLPAFPREKLTLRLLLGSGAFGEVYE GTAVDILGVGSGEIKVAVKTLKKGSTDQ EKIEFLKEAHLMSKFNHPNILKQLGVCLL NEPQYIILELMEGGDLLTYLRKARMATF YGPLLTLVDLVDLCVDISKGCVYLERMH FIHRDLAARNCLVSVKDYTSPRIVKIGDF GLARDIYKNDYYRKRGEGLLPVRWMAP ESLMDGIFTTQSDVWSFGILIWEILTLGH QPYPAHSNLDVLNYVQTGGRLEPPRNC PDDLWNLMTQCWAQEPDQRPTFHRIQ DQLQLFRNFFLNSIYKSR	COC1=CC2=C(C=CN=C2C=C1OC)O C3=CC=C(C=C3)NC(=O)C4(CC4)C(= O)NC5=CC=C(C=C5)F

Supplementary MDP files

I)Energy minimization

```
; minim.mdp - used as input into grompp to generate em.tpr
; Parameters describing what to do, when to stop and what to save
integrator  = steep         ; Algorithm (steep = steepest descent minimization)
emtol       = 1000.0        ; Stop minimization when the maximum force < 1000.0 kJ/mol/nm
emstep      = 0.01          ; Minimization step size
nsteps      = 50000         ; Maximum number of (minimization) steps to perform

; Parameters describing how to find the neighbors of each atom and how to calculate the interactions
nstlist         = 1         ; Frequency to update the neighbor list and long range forces
cutoff-scheme   = Verlet    ; Buffered neighbor searching
ns_type         = grid      ; Method to determine neighbor list (simple, grid)
coulombtype     = PME       ; Treatment of long range electrostatic interactions
rcoulomb        = 1.0       ; Short-range electrostatic cut-off
rvdw            = 1.0       ; Short-range Van der Waals cut-off
pbc             = xyz       ; Periodic Boundary Conditions in all 3 dimensions
```

II)NVT ensemble

```
title                   = NVT equilibration
define                  = -DPOSRES  ; position restrain the protein
; Run parameters
integrator              = md        ; leap-frog integrator
nsteps                  = 100000    ; 2 * 100000 = 200 ps
dt                      = 0.002     ; 2 fs
; Output control
nstxout                 = 500       ; save coordinates every 1.0 ps
nstvout                 = 500       ; save velocities every 1.0 ps
nstenergy               = 500       ; save energies every 1.0 ps
nstlog                  = 500       ; update log file every 1.0 ps
; Bond parameters
continuation            = no        ; first dynamics run
constraint_algorithm    = lincs     ; holonomic constraints
constraints             = h-bonds   ; bonds involving H are constrained
lincs_iter              = 1         ; accuracy of LINCS
lincs_order             = 4         ; also related to accuracy
; Nonbonded settings
cutoff-scheme           = Verlet    ; Buffered neighbor searching
ns_type                 = grid      ; search neighboring grid cells
nstlist                 = 10        ; 20 fs, largely irrelevant with Verlet
rcoulomb                = 1.0       ; short-range electrostatic cutoff (in nm)
rvdw                    = 1.0       ; short-range van der Waals cutoff (in nm)
DispCorr                = EnerPres  ; account for cut-off vdW scheme
; Electrostatics
coulombtype             = PME       ; Particle Mesh Ewald for long-range electrostatics
pme_order               = 4         ; cubic interpolation
fourierspacing          = 0.16      ; grid spacing for FFT
```

```
; Temperature coupling is on
tcoupl                = V-rescale           ; modified Berendsen thermostat
tc-grps               = Protein Non-Protein   ; two coupling groups - more accurate
tau_t                 = 0.1     0.1          ; time constant, in ps
ref_t                 = 300     300          ; reference temperature, one for each group, in K
; Pressure coupling is off
pcoupl                = no       ; no pressure coupling in NVT
; Periodic boundary conditions
pbc                   = xyz       ; 3-D PBC
; Velocity generation
gen_vel               = yes       ; assign velocities from Maxwell distribution
gen_temp              = 300       ; temperature for Maxwell distribution
gen_seed              = -1        ; generate a random seed
```

II)NPT ensemble

```
title                 = OPLS Lysozyme NPT equilibration
define                = -DPOSRES  ; position restrain the protein
; Run parameters
integrator            = md        ; leap-frog integrator
nsteps                = 100000    ; 2 * 100000 = 200 ps
dt                    = 0.002     ; 2 fs
; Output control
nstxout               = 500       ; save coordinates every 1.0 ps
nstvout               = 500       ; save velocities every 1.0 ps
nstenergy             = 500       ; save energies every 1.0 ps
nstlog                = 500       ; update log file every 1.0 ps
; Bond parameters
continuation          = yes       ; Restarting after NVT
constraint_algorithm    = lincs     ; holonomic constraints
constraints           = h-bonds   ; bonds involving H are constrained
lincs_iter            = 1         ; accuracy of LINCS
lincs_order           = 4         ; also related to accuracy
; Nonbonded settings
cutoff-scheme         = Verlet    ; Buffered neighbor searching
ns_type               = grid      ; search neighboring grid cells
nstlist               = 10        ; 20 fs, largely irrelevant with Verlet scheme
rcoulomb              = 1.0       ; short-range electrostatic cutoff (in nm)
rvdw                  = 1.0       ; short-range van der Waals cutoff (in nm)
DispCorr              = EnerPres  ; account for cut-off vdW scheme
; Electrostatics
coulombtype           = PME       ; Particle Mesh Ewald for long-range electrostatics
pme_order             = 4         ; cubic interpolation
fourierspacing        = 0.16      ; grid spacing for FFT
; Temperature coupling is on
tcoupl                = V-rescale           ; modified Berendsen thermostat
tc-grps               = Protein Non-Protein   ; two coupling groups - more accurate
tau_t                 = 0.1     0.1          ; time constant, in ps
ref_t                 = 300     300          ; reference temperature, one for each group, in K
; Pressure coupling is on
```

```
pcoupl                  = Parrinello-Rahman     ; Pressure coupling on in NPT
pcoupltype              = isotropic             ; uniform scaling of box vectors
tau_p                   = 2.0                   ; time constant, in ps
ref_p                   = 1.0                   ; reference pressure, in bar
compressibility         = 4.5e-5                ; isothermal compressibility of water, bar^-1
refcoord_scaling        = com
; Periodic boundary conditions
pbc                     = xyz       ; 3-D PBC
; Velocity generation
gen_vel                 = no        ; Velocity generation is off
```

IV)Production MD

```
title                   =  ROS1 Production run 600ns
; Run parameters
integrator              = md        ; leap-frog integrator
nsteps                  = 300000000   ; 2 * 300000000 = 600000 ps (600 ns)
dt                      = 0.002     ; 2 fs
; Output control
nstxout                 = 0         ; suppress bulky .trr file by specifying
nstvout                 = 0         ; 0 for output frequency of nstxout,
nstfout                 = 0         ; nstvout, and nstfout
nstenergy               = 5000      ; save energies every 10.0 ps
nstlog                  = 5000      ; update log file every 10.0 ps
nstxout-compressed      = 5000      ; save compressed coordinates every 10.0 ps
compressed-x-grps       = System    ; save the whole system
; Bond parameters
continuation            = yes       ; Restarting after NPT
constraint_algorithm    = lincs     ; holonomic constraints
constraints             = h-bonds   ; bonds involving H are constrained
lincs_iter              = 1         ; accuracy of LINCS
lincs_order             = 4         ; also related to accuracy
; Neighborsearching
cutoff-scheme           = Verlet    ; Buffered neighbor searching
ns_type                 = grid      ; search neighboring grid cells
nstlist                 = 10        ; 20 fs, largely irrelevant with Verlet scheme
rcoulomb                = 1.0       ; short-range electrostatic cutoff (in nm)
rvdw                    = 1.0       ; short-range van der Waals cutoff (in nm)
; Electrostatics
coulombtype             = PME       ; Particle Mesh Ewald for long-range electrostatics
pme_order               = 4         ; cubic interpolation
fourierspacing          = 0.16      ; grid spacing for FFT
; Temperature coupling is on
tcoupl                  = V-rescale             ; modified Berendsen thermostat
tc-grps                 = Protein Non-Protein   ; two coupling groups - more accurate
tau_t                   = 0.1     0.1           ; time constant, in ps
ref_t                   = 300     300           ; reference temperature, one for each group, in K
; Pressure coupling is on
pcoupl                  = Parrinello-Rahman     ; Pressure coupling on in NPT
pcoupltype              = isotropic             ; uniform scaling of box vectors
```

```
tau_p                  = 2.0                  ; time constant, in ps
ref_p                  = 1.0                  ; reference pressure, in bar
compressibility        = 4.5e-5               ; isothermal compressibility of water, bar^-1
; Periodic boundary conditions
pbc                    = xyz       ; 3-D PBC
; Dispersion correction
DispCorr               = EnerPres  ; account for cut-off vdW scheme
; Velocity generation
gen_vel                = no        ; Velocity generation is off
```
